# Prevention of the Progression of lupus Nephritis in MRL/lpr Mice by Modulating miR-9-5p/Foxo1 Axis

**DOI:** 10.33549/physiolres.935539

**Published:** 2025-08-01

**Authors:** Huimin YAN, Wei TONG

**Affiliations:** 1 Department of Nephrology, Jiujiang City Key Laboratory of Cell Therapy, JiuJiang NO.1 People’s Hospital, Jiujiang, China

**Keywords:** Foxo1, *lupus nephritis*, miR-9-5p, MRL/lpr mice

## Abstract

MiR-9-5p is up-regulated in lupus nephritis (LN) patients and targets Foxo1 that is a protective factor against renal disorders. In the current study, the role of miR-9-5p/Foxo1 LN progression was assessed and the associated mechanism was explored. The levels of LN-associated miRs were firstly detected in MRL/lpr mice. Then the effect of miR-9-5p modulation on the viability of SV40 MES 13 cells was detected. MRL/lpr mice were treated with miR agomirs or antagonists, and effects on renal structure and function were assessed. MiR-9-5p was selected as the potential target, which was up-regulated in MRL/lpr mice, contributing to the suppressed expression of Foxo1. The modulation of miR-9-5p *in vitro* influenced the viability of SV40 MES 13 cells. The progression of LN in mice was also associated with the increased level of miR-9-5p and the decreased level of Foxo1. The administration of miR agomirs significantly impaired renal structure and function impairments associated with LN, along with the suppressed expression of Foxo1, while antagonists improved these features by up-regulating Foxo1 level. The current study demonstrated that miR-9-5p showed LN promoting effects, which depended on the inhibition of Foxo1.

## Introduction

Systemic lupus erythematosus (SLE) is a chronic autoimmune disease affecting numerous organs and tissues, and the involvement of kidney is one of the most significant predictors of morbidity and mortality of SLE. Being defined as lupus nephritis (LN), the disorder occurs in up to 60 % of all the SLE patients and worse still, about 10–30 % of LN patients with class III–V will progress into end stage kidney disease (ESKD) within 15 years after diagnosis [1–6]. Currently, the major treatment strategies for LN include steroids, immunosuppressive agents, B-cell modulating therapies or calcineurin inhibitors [7]. According to recent investigations, the 10-year-survival probability of LN patients has reached 88 % with the treatment of immunosuppressor [8,9]. However, the optimal therapy varies among LN patients and conventional drugs are also associated with marked toxicity. Currently, with our improved understanding of the immunopathogenesis of SLE, novel immunological targeted therapeutics are being characterized and provided, including immunotherapy with haematopoietic stem cell rescue, anti-cytokine medicines, complement directed treatments, and both B and T cell directed therapies [10]. Given the diverse options for anti-LN therapies, novel LN treatments should be at least as effective as and less harmful than current treatments.

Prolonged inflammation plays an important role in the development of kidney dysfunction, and thus, the inhibition of response has long been regarded as a promising strategy for handling renal diseases [11,12]. Recently, emerging evidence shows that miRs are closely involved in the modulation of innate and adaptive immune responses [13]. Based on the study by Vinuesa *et al*., different target sites for over 140 conserved miRs exist in lupus susceptibility genes, including those involved in the modulation of inflammation responses [14,15]. Regarding the role of miRs in LN, miR-145 regulates LN progression via CSF1 mediated JAK/STAT signaling pathway and employed as a new therapeutic target for LN treatment [16]. Moreover, miR-30a directly regulates the function Notch1 and alleviates podocyte injury in LN [17]. Except for miRs playing protective effects, miRs such as miR-21 and miR-15a are associated with the progression of LN [18]. Thus, the exploration of the potential miRs that can serve as immune treatment targets of LN will provide valuable information for the future management of the disease.

To determine the changes in miR profiles in the progression of LN, MRL/lpr mice were employed as the representative model. The change in miR expression profile was firstly determined. Then the kidney function and structure under the modulation of the select target, i.e., miR-9-5p, were assessed. Additionally, the current study also attempted to explore the downstream pathways involved in the function of miR-9-5p. As reported by Ermakov *et al*., the miR has been reported to play a key role in the progression of LN [19] and the downstream effector of miR-9-5p, Foxo1 plays a renal protective role in the progression of diverse renal disorders [20]. Compared with the previous published studies [14,16–19], the current study firstly performed a comprehensive exploration regarding the dys-expressed miRs associated with LN progression, and then selected miR-9-5p/Foxo1 axis as the target, which provide additional information for the future development of handling strategies for LN.

## Materials and Methods

### Microarray detection

Nine-week old female MRL/lpr mice and MRL/MPJ mice were purchased from Shanghai SLAC Laboratory Animal Co., Ltd (China) and housed in 25±1˚C with a constant humidity of 45–55 % with food and water available for eight weeks. Upon completion of house, mice were sacrificed using overdose pentobarbital sodium (200 mg/kg) for the collection of kidney samples. All the animal assays were performed following the approval of ethic committee of JiuJiang NO.1 People’s Hospital and the ethical standards in the 1964 Declaration of Helsinki and its later amendments.

The miR expression profiles in kidney tissues were determined with microarray detection. Total RNA was extracted was extracted using a miRNeasy Kit and a mirVana miRNA Isolation Kit, respectively. Following labeling with a miRNA Complete Labeling and Hyb Kit, 100 ng of RNA were sequenced on the Illumina NextSeq 500 platform using the miRDeep2 database as a guide. After being normalized, the output data was entered into Excel spreadsheets that held the profiles of microRNA expression.

### Bioinformatic analysis

Based on the criterion of |log2 fold change (FC)| > 2 and an adjusted P-value<0.05, differentially expressed miRs were identified. In order to identify the highly sensitive miRs associated with gefitinib resistance, RT-qPCR detection with PC9R cells were used to further evaluate the expression status of the eight most substantially altered miRs among the upregulated or downregulated miRs. Afterwards, the potential targets of 8 differentially expressed miRs (miR-145, miR-155, miR-30a, miR-2861, miR-1915-3p, miR-21, miR-9-5p, miR-15a) were predicted using TargetScan7.1 and miRBase Targets Release Version v5 [21–23].

### Reverse transcription quantitative PCR (RT-qPCR)

Total RNA in cells or tissue samples was extracted using the Trizol technique (Thermo, USA) and RNA concentration was evaluated using NANO 2000. cDNA templates were produced with the help of Super M-MLV Reverse Transcriptase (cat. no. PR6502, BioTeke, China). Real-time PCR was then performed on cDNA templates using a reaction system of 20 μl, 1 μl template, 0.5 μl of each primer (miR-9-5p, forward: 5′-TGCGCTCTTTGGTTATCTAGCTG-3′, reverse, 5′-CCAGTGCAGGGTCCGAGGTATT-3′; U6, forward: 5′-CGCAAGGATGACACGCAAAT-3′, reverse: 5′-GTGCAGGGTCCGAGGTATTC-3′), 10 μl 2× Power Taq PCR MasterMix (BioTeke, China), and 8 μl ddH_2_O. The following conditions were used to execute the amplifications: a denaturation stage at 94 °C for 2 min, 40 cycles of amplification at 94 °C for 15 s, 60 °C for 15 s, and 72 °C for 15 s, followed by signal scanning. The relative expression levels of miR-9-5p were determined using the 2^−ΔΔct^ formula.

### Dual luciferase assay

The interaction between Foxo1 and miR-9-5p was verified with a dual luciferase assay. Wild type (WT) and mutant type (MUT) of Foxo1 3′UTR, and mimics of miR-9-5p (5′-TCTTTGGTTATCTAGCTGTATGAAA-3′) and negative control (NC) mimics (5′-UUCUCCGAACGUGUCACGUTT-3′) were trans-fed into HEK293 cells using Liposome 3000 (Invitrogen, USA) according to the manufacturer’s instruction with renilla luciferase as the internal control. The changes in luciferase activity was detected 24 h after the transfection using Luciferase Activity Detection Kit (Promega, USA) and a Chemiluminescence Apparatus (Lumat LB 9507, Berthold, German).

### Cell culture and grouping

Murine glomerular mesangial cell SV40 MES 13 and human embryonic kidney cells HEK293 (Shanghai, China) were acquired from Shanghai Zhong Qiao Xin Zhou Biotechnology Co.,Ltd. And cultured routinely in an atmosphere of 5 % CO_2_ and 95 % air in MEM supplemented with 5 % FBS. Afterwards, SV40 MES 13 cells were incubated with various doses of TGF-1 to simulate fibrosis *in vitro* to confirm the likelihood that LN-associated fibrosis might stimulate the expression of miR-9-5p: recombinant murine TGF-1 (rmTGF-1) was administered to SV40 MES 13 cells at 0.10 ng/ml for 12 h. To detected the role of miR-9-5p in the development of LN, the expression level of miR-9-5p were modulated with specific mimics (5′-TCTTTGGTTATCTAGC TGTATGAAA-3′) and inhibitors (Synthesized by Sango Biotch., Shanghai, China) 12 h before rmTGF-1 treatment. Upon completion of the treatment, the cells were the collected for subsequent tests.

### CCK-8 assay

The viability of SV40 MES 13 cells was assessed using the CCK-8 method: cells (1 × 10^5^ cells/well) in different groups were collected every 24 h and incubated with 10 μl of CCK-8 solution at 37 °C for another 2 h. The absorbance was analyzed at 450 nm using a Microplate Reader (Molecular Devices, San Jose, CA, USA).

### Western blotting

RIPA lysis buffer (Beyotime Biotechnology, China) was used to lyse kidney tissues, and the BCA Protein Concentration Kit was used to measure protein concentrations (Beyotime Biotechnology, China). After separated by sodium dodecyl sulfatepolyacrylamide gel electrophoresis (SDS-PAGE) at 80V for 2.5 h, the proteins were transferred to membranes and then incubated with primary antibodies against Foxo1 (1:1000) (Cell Signaling Technology, USA) and GAPDH (1:500) (Bioss, China) at 4˚C overnight. The membranes were then treated with secondary IgG-HRP antibodies (1:5000). ECL Plus reagent (Beyotime Biotechnology, China) was used to create protein bands. The relative expression levels of the proteins (Media Cybernetics, USA) were analyzed with a Gel-Pro-Analyzer.

### Animals and administration

The results of *in vitro* assays were then verified with mice, and 24 mice were divided into four groups (six for each group). Control group, MRL/lpr mice were orally administrated with saline for eight weeks. Agomir group, MRL/MPJ mice received tail injection of miR-9-5p agomir once a week for eight weeks. Antagonist group, MRL/lpr mice received tail injection of miR-9-5p antagonist once a week for eight weeks. Upon completion of house, mice were sacrificed for the collection of blood, urine, spleen, and kidney samples.

### Renal function and detection

The alternatives in renal functions were evaluated by detecting the levels of 24-hour proteinuria, blood urea nitrogen (BUN), and serum creatinine using detection kits (Nanjing Jiancheng Bioengineering Institute, China) according to the manufacturer’s instructions. The production of TGF-β1 (Multi Sciences, China) in kidney tissues and anti-dsDNA (Biovision, USA) in serum samples were determined using ELISA kits according to the manufacturer’s instructions.

### Histological staining

For H&E staining, tissues were dehydrated with different concentration of alcohol, embedded in paraffin, sectioned, and stained with H&E. The results were detected under a microscope (BX53, Olupums, Japan) at a 200× magnification.

For Masson staining, kidney tissues were stained with three different dyes: firstly, tissues were incubated in hematoxylin solution for six minutes, and then with ponceau and acid fuchsin solution for one minute, and finally phosphomolybdic acid solution for five minutes. A microscope (BX53, Olumpus, Japan) was used to take the pictures at a magnification of 200x: collagen fibers were stained blue, while muscle fibers were stained red.

For PAS staining, kidney samples were dehydrated, vitrified, and then cut into 5-μm thickness and stained with PAS solution for 15 min. Afterwards, sections were re-stained with hematoxylin for 2 min and incubated with different concentrations of alcohol. The results were detected under a microscope (BX53, Olupums, Japan) at 200× magnification.

### Statistical analysis

Data were represented as mean ± standard deviation (SD). One-way analysis of variance (ANOVA), student t test, and post hos analysis using Tukey method were performed using GraphPad Prism version 6.0 (GraphPad Software, Inc., San Diego, CA) with a significant level of 0.05 (two-tailed P value).

## Results

### Analysis of miR expression profiling associated with LN progression

**Fig. 1:**
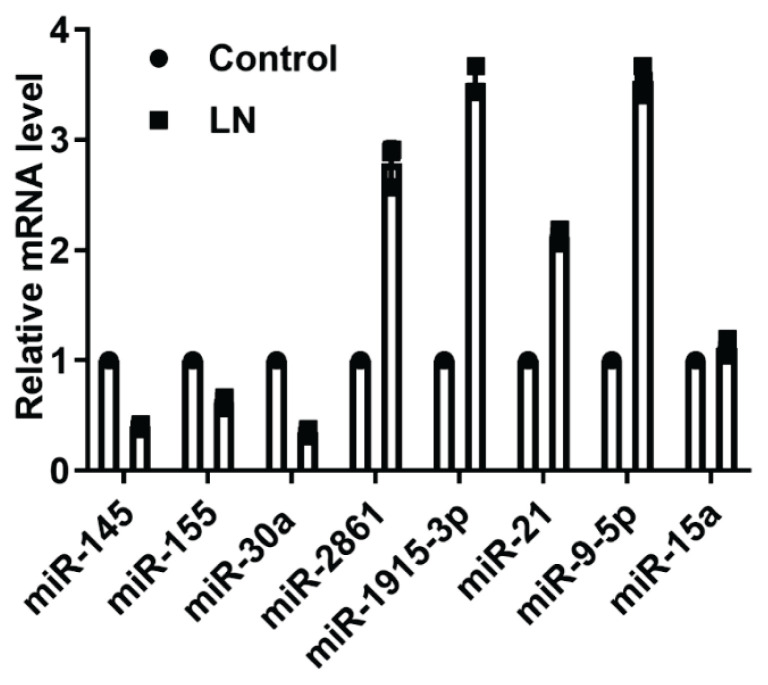
Detection of miR expression profile in kidney tissues of MRL/lpr mice and MRL/MPJ mice. The levels of miR-145, miR-155, miR-30a, miR-2861, miR-1915-3p, miR-21, miR-9-5p, and miR-15a were detected with RT-qPCR. Each assay was represented by three replicates.

To clarify the role of miR in the progression of LN, the expression profile of miRs in kidney tissues in LN mice was detected in comparison to healthy mice. After the data normalization, a total of 352 dys-expressed miRs was identified in LN mice, including 108 up-regulated miRs and 244 down-regulated miRs (Table S1 and Table S2). To validate the results of microarray detection, the expression status of eight most substantially altered miRs was detected using RT-qPCR. As shown in Fig. 1, the levels of miR-145, miR-155, and miR-30a were suppressed MRL/lpr mice compared with MRL/MPJ mice, while the levels of miR-2861, miR-1915-3p, miR-21, and miR-9-5p were induced. The level of miR-15a was influenced by the progression of LN. Based on the comparison of P value and fold-change, miR-9-5p was selected as the potential target for underlying the pathogenesis of LN due to its relatively high fold-change and small P value.

### MiR-9-5p directly interacted with encoding gene of Foxo1 in PC9 cells

**Fig. 2:**
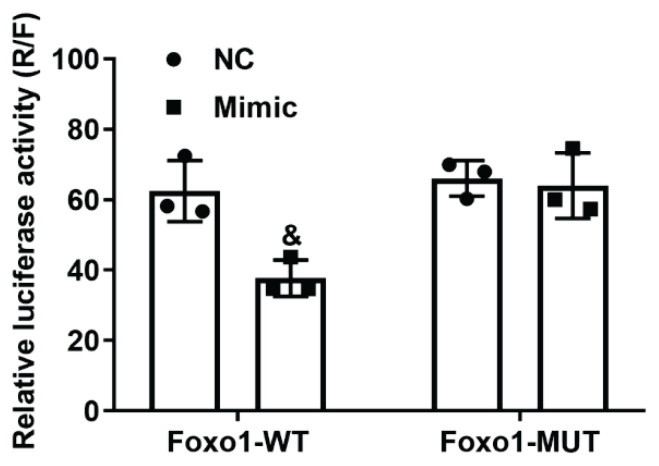
MiR-9-5p direclty interacts with the 3′UTR sequence of Foxa1. The interaction was detected using a dual luciferase assay. & P<0.05 vs. NC group.

To confirm that miR-9-5p could directly regulate the function of Foxo1 in kidney cells, the interaction between miR-9-5p and Foxo1 was detected using a dual luciferase assay in HEK293 cells. As shown in Fig. 2, the relative luciferase activity in HEK293 cells could only be decreased by co-transfection with miR-9-5p mimics and WT Foxo1 3′UTR sequence, indicating that miR-9-5p could bind to the Foxo1 3′UTR region selectively. All things considered, it seemed sense to infer that miR-9-5p directly controls Foxo1 function in HEK293 cells.

### MiR-9-5p was involved in the LN progression by inhibiting Foxo1 expression

**Fig. 3:**
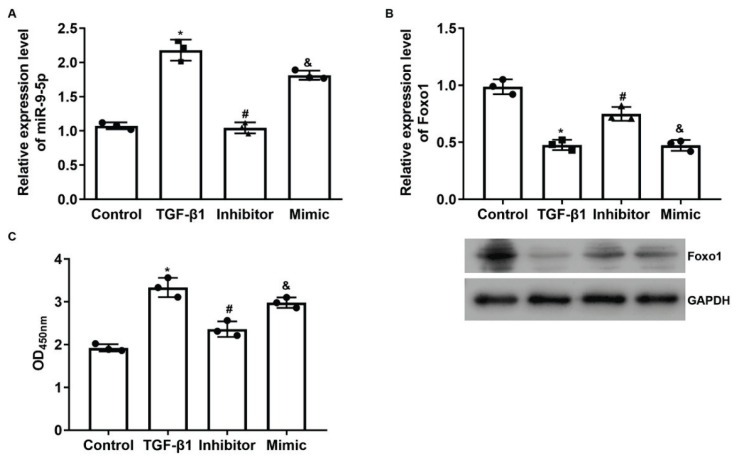
Modulation of miR-9-5p level influenced the viability and activity of Foox1 in SV40 MES 13 cells. The level of miR-9-5p was modulated with specific mimics and inhibitors in SV40 MES 13 cells co-treated with TGF-β1. **A)** quantitative analysis results of RT-qPCR detection of miR-9-5p. **B)** quantitative analysis results and representative images of western blotting detection of Foxo1. **C)** quantitative analysis results of CCK-8 detection of cell viability. * *P*<0.05 vs. Control group. # *P*<0.05 vs. TGF-β1 group. & *P*<0.05 vs. inhibitor group. Each assay was represented by three replicates.

To verify the key role of miR-9-5p/Foxo1 axis in the progression of LN, the level of miR-9-5p was modulated with specific mimics and inhibitors in SV40 MES 13 cells. Then the *in vitro* renal injury model was induced with TGF-β1. As shown in Fig. 3A, TGF-β1 increased the expression of miR-9-5p, while suppressed the expression of Foxo1 (Fig. 3B), which contributed to the increased viability of SV40 MES 13 cells (Fig. 3C). The increased viability of SV40 MES 13 cells implied the initiation of a fibrotic process induced by TGF-β1, which was then reversed by miR-9-5p inhibitor. However, for cells treated with miR-9-5p mimics, the fibrotic process was also initiated (Fig. 3). The data showed that miR-9-5p promoted LN progression by inhibiting Foxo1 expression.

### Effects of miR-9-5p modulation on the renal structure in MRL/lpr mice

**Fig. 4:**
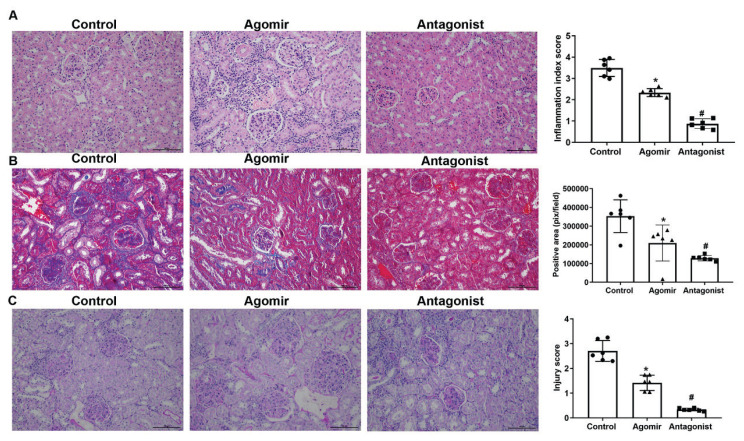
Modulation of miR-9-5p influences structure integrity associated with LN progression. Kidney tissues were collected from 17-week-old MRL/lpr mice, MRL/MPJ mice treated with miR-9-5p agomir, and MRL/lpr mice treated with miR-9-5p antagonist. **A)** representative images and quantitative analysis result of H&E staining of kidney tissues. **B)** representative images and quantitative analysis result of Masson staining of kidney tissues. **C)** representative images and quantitative analysis result of PAS staining of kidney tissues. * *P*<0.05 vs. Control group. # *P*<0.05 vs. Agomir group. Scale bar, 100 μm. Each assay was represented by six replicates.

The potential role of miR-9-5p were then verified in mice models by detecting changes in renal histology with H&E staining, Masson, and PAS staining. Compared with control mice, the destruction in renal tissues was weaker in MRL/MPJ mice injected with miR-9-5p agomirs (Fig. 4A), which was associated with the less deposition of collagens (Fig. 4B) and glycogens (Fig. 4C). The impairments on renal structures were attenuated by the miR-9-5p antagonist. In MRL/lpr mice administrated with the antagonist, the tissues destruction was attenuated, and the deposition of collagens and glycogens was inhibited (Fig. 4) compared with Control group, solidly indicating the protective effects of miR-9-5p antagonist against LN.

### Effects of miR-9-5p modulation on the renal function in MRL/lpr mice

**Fig. 5:**
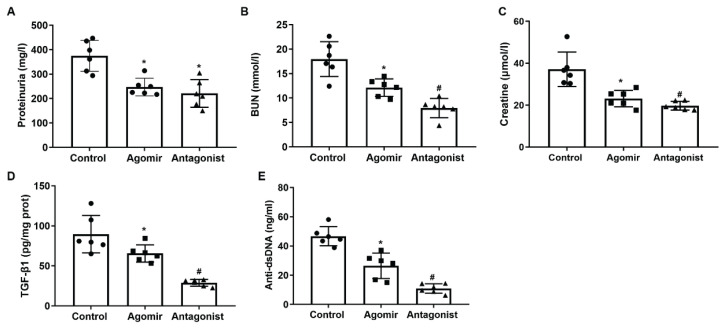
Modulation of miR-9-5p influences renal dysfunction associated with LN progression. Kidney tissues and serum samples were collected from 17-week-old MRL/lpr mice, MRL/MPJ mice treated with miR-9-5p agomir, and MRL/lpr mice treated with miR-9-5p antagonist. **A)** quantitative analysis result of ELISA detection of 24-hour proteinuria. **B)** quantitative analysis result of ELISA detection of BUN. **C**, quantitative analysis result of ELISA detection of serum creatinine. **D)** quantitative analysis result of kidney ELISA detection of TGF-β1. **E)** quantitative analysis result of ELISA detection of serum dsDNA. * *P* < 0.05 vs. Control group. # *P* < 0.05 vs. Agomir group. Scale bar, 100 μm. Each assay was represented by six replicates.

The effects of miR-9-5p modulation on the renal function of LN mice were assessed by measuring the levels of 24-h proteinuria, BUN, and serum creatinine. The values of the three indicators were firstly induced in MRL/lpr mice and MRL/MPJ injected with miR-9-5p agomirs with the progression of LN, but was then significantly improved in the MRL/lpr mice administrated with miR-9-5p antagonist (Fig. 5A–5C). The renal level of TGF-β1 and the serum level of anti-dsDNA in the mice were also detected. The data showed that the level of TGF-β1 were increased in mice developing LN symptoms (Fig. 5D and 5E). The administration of miR-9-5p antagonist also restored the expression status of these indicators (Fig. 5D and 5E), further implying the improving effects of miR-9-5p inhibition on renal function of LN mice.

### Effects of miR-9-5p modulation on the expression of Foxo1

**Fig. 6:**
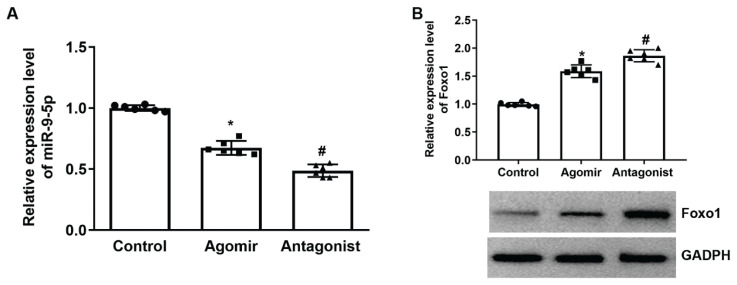
Modulation of miR-9-5p influenced the level of Foxo1 in kideny tissues of mice. Kidney tissues were collected from 17-week-old MRL/lpr mice, MRL/MPJ mice treated with miR-9-5p agomir, and MRL/lpr mice treated with miR-9-5p antagonist. **A)** quantitative analysis results of RT-qPCR detection of miR-9-5p. **B)** quantitative analysis results and representative images of western blotting detection of miR-9-5p. * P < 0.05 vs. Control group. # P < 0.05 vs. Agomir group. Scale bar, 100 μm. Each assay was represented by six replicates.

The expression change in Foxo1 under different treatments was assessed with RT-qPCR and western blotting detections in renal tissue. Along with the increase in miR-9-5p level in the MRL/lpr mice and MRL/MPJ injected with miR-9-5p agomirs, the expression of Foxo1 decreased with the development of LN in kidney tissues (Fig. 6). After the inhibition of miR-9-5p, the level of Foxo1 were restored (Fig. 6). The data verified the interaction between miR-9-5p and Foxo1 as detected *in vitro* models.

## Discussion

The current study provides robust evidence for the pivotal role of the miR-9-5p/Foxo1 axis in the pathogenesis and progression of LN. By analyzing the interaction between miR-9-5p and Foxo1, we demonstrated how miR-9-5p directly regulates Foxo1 expression, contributing to the progression of LN. The data showed that miR-9-5p inhibitors or antagonists attenuated LN-induced renal tissue destruction and collagen deposition as well as suppressing the production of LN indicators, solidly showing a treatment effect against LN.

Our findings shed light on the critical molecular mechanisms underlying LN and highlight the therapeutic potential of targeting this axis to alleviate disease symptoms and improve renal outcomes.

The pathological changes of LN are driven by dysregulated of many miRs. Among these, miR-9-5p has emerged as a significant player due to its elevated expression in LN models and its role in regulating genes involved in inflammation and fibrosis [14,19]. In the current study, we observed that miR-9-5p is significantly upregulated in LN mouse models and in TGF-β1-treated mesangial cells, both of which are well-established systems for studying LN pathogenesis [16,17]. LN, as a severe complication of systemic lupus erythematosus, is characterized by inflammation, fibrosis, and cellular proliferation within the kidney [24]. The activation of neutrophils can contribute to the pathogenesis of SLE and LN, which is preceded an increase in IFN and plasmablast-related transcript. Therefore, the suppression of immune response of LN patients has been conceived as a potential treatment strategy. The dead neutrophils will release neutrophil extracellular traps (NETs) which is normally a host defense mechanism to trap and kill microorganisms. However, in SLE patients, especially those with LN, the formation of NET will form a source of nuclear antigens [25].

The mechanism driving the function of miR-9-5p was then explored by detecting the changes in miR-9-5p/Foxo1 axis. The level of miR-9-5p was both up-regulated in LN mice and TGF-β1-treated glomerular mesangial cells, and our data demonstrated that miR-9-5p promotes LN progression by targeting and down-regulating Foxo1, a key renal protective factor. Foxo1 has been widely studied for its role in maintaining cellular homeostasis, regulating inflammation, and inhibiting fibrosis in various tissues, including the kidney [20]. The current study performed a dual luciferase assay to verity the direct interaction between miR-9-5p and Foxo1 in kidney, confirming the potential involvement of miR-9-5p/Foxo1 in the progression of LN-related symptoms. This interaction significantly reduces Foxo1 expression, which was associated with enhanced mesangial cell proliferation and increased deposition of extracellular matrix proteins, both hallmarks of LN [18,20]. The therapeutic potential of modulating the miR-9-5p/Foxo1 axis was further supported by intervention studies. Using miR-9-5p inhibitors in both *in vitro* and *in vivo* models, it is demonstrated that suppression of miR-9-5p effectively restores Foxo1 expression, thereby reversing LN-associated pathological changes. In MRL/lpr mice, a well-established model of LN, miR-9-5p inhibition resulted in significant improvements in renal structure and function. Histological analysis revealed decreased collagen and glycogen deposition, along with restoration of normal renal architecture. Moreover, biochemical markers of renal function, such as serum creatinine, BUN, and proteinuria, were normalized following miR-9-5p inhibition [13,26]. These results highlight the therapeutic efficacy of targeting miR-9-5p: the comprehensive exploration of the miR and its downstream effector will not only provide valuable information for the early diagnosis of LN, but will also serve as a target for the treatment of LN. Thus, future work focusing on the detailed explanation regarding the signaling transduction and investigation regarding the *in vivo* of level mir-9-5p is needed to promote the clinical application of the miR-related pathways. However, some limitations should be acknowledged. While we established a clear relationship between miR-9-5p and Foxo1 in LN models, the broader implications of this axis in other renal disorders remain to be explored. Furthermore, the potential interactions between miR-9-5p and other signaling pathways involved in LN, such as PI3K/Akt and TGF-β1, warrant further investigation [26]. Understanding these complex interactions will be critical for developing more effective and targeted therapies.

In conclusion, the current study highlights the critical role of the miR-9-5p/Foxo1 axis in LN pathogenesis. By suppressing Foxo1 expression, miR-9-5p drives key pathological changes, including mesangial cell proliferation and fibrosis, which contribute to renal damage. Modulation of this axis through miR-9-5p inhibitors restores Foxo1 activity and ameliorates LN symptoms, providing a novel therapeutic strategy for managing this severe condition. Our findings pave the way for further research into miRNA-based therapies and underscore the potential of the miR-9-5p/Foxo1 axis as both a therapeutic target and biomarker for LN.

## Supplementary Information





## References

[1] Maroz N, Segal MS (2013). Lupus nephritis and end-stage kidney disease. Am J Med Sci.

[2] Ward MM (2000). Changes in the incidence of end-stage renal disease due to lupus nephritis, 1982–1995. Arch Intern Med.

[3] Okpechi IG, Ameh OI (2015). Lupus nephritis: An approach to diagnosis and treatment in South Africa. S Afr Med J.

[4] Tang Y, Zhang X, Ji L, Mi X, Liu F, Yang L, Qin W (2015). Clinicopathological and outcome analysis of adult lupus nephritis patients in China. Int Urol Nephrol.

[5] Hanly JG, O’Keeffe AG, Su L, Urowitz MB, Romero-Diaz J, Gordon C, Bae SC, Bernatsky S, Clarke AE (2016). The frequency and outcome of lupus nephritis: results from an international inception cohort study. Rheumatology (Oxford).

[6] Balajkova V, Olejarova M, Moravcova R, Kozelek P, Posmurova M, Hulejova H, Senolt L (2020). Is serum TWEAK a useful biomarker of neuropsychiatric systemic lupus erythematosus?. Phys Res.

[7] Dall’Era M (2017). Treatment of lupus nephritis: current paradigms and emerging strategies. Curr Opin Rheumatol.

[8] Imran TF, Yick F, Verma S, Estiverne C, Ogbonnaya-Odor C, Thiruvarudsothy S, Reddi AS, Kothari N (2016). Lupus nephritis: an update. Clin Exp Nephrol.

[9] Cervera R, Khamashta MA, Font J, Sebastiani GD, Gil A, Lavilla P, Mejía JC, Aydintug AO, Chwalinska-Sadowska H (2003). Morbidity and mortality in systemic lupus erythematosus during a 10-year period: a comparison of early and late manifestations in a cohort of 1,000 patients. Medicine (Baltimore).

[10] Schieppati A, Remuzzi G (2008). Novel therapies of lupus nephritis. Curr Opin Nephrol Hypertens.

[11] Lee SB, Kalluri R (2010). Mechanistic connection between inflammation and fibrosis. Kidney Int Suppl.

[12] Yuan XK, Ni PS, Yan ZH, Yu Z, Wang ZZ, Zhang CK, Li FH, Yu XM (2024). Effects of life-long exercise on age-related inflammation, apoptosis, oxidative stress, ferroptosis markers, and NRF2/KAEP 1/Klotho Pathway in Rat Kidneys. Physiol Res.

[13] Tili E, Michaille JJ, Calin GA (2008). Expression and function of micro-RNAs in immune cells during normal or disease state. Int J Med Sci.

[14] Vinuesa CG, Rigby RJ, Yu D (2009). Logic and extent of miRNA-mediated control of autoimmune gene expression. Int Rev Immunol.

[15] Mo W, Jin J, Wang X, Luan W, Yan J, Long X (2023). MicroRNA-206 Contributes to the Progression of Preeclampsia by Suppressing the Viability and Mobility of Trophocytes via the Inhibition of AGTR1. Physiological research.

[16] Liao W, He XJ, Zhang W, Chen YL, Yang J, Xiang W, Ding Y (2022). MiR-145 participates in the development of lupus nephritis by targeting CSF1 to regulate the JAK/STAT signaling pathway. Cytokine.

[17] Guo A, Sun Y, Xu X, Xing Q (2022). MicroRNA-30a Targets Notch1 to Alleviate Podocyte Injury in Lupus Nephritis. Immunol Invest.

[18] Chafin CB, Reilly CM (2013). MicroRNAs implicated in the immunopathogenesis of lupus nephritis. Clin Dev Immunol.

[19] Ermakov EA, Kabirova EM, Sizikov AE, Buneva VN, Nevinsky GA (2020). IgGs-Abzymes from the Sera of Patients with Systemic Lupus Erythematosus Hydrolyzed miRNAs. J Inflamm Res.

[20] Hong YA, Bae SY, Ahn SY, Kim J, Kwon YJ, Jung WY, Ko GJ (2017). Resveratrol Ameliorates Contrast Induced Nephropathy Through the Activation of SIRT1-PGC-1α-Foxo1 Signaling in Mice. Kidney Blood Press Res.

[21] Kanehisa M, Sato Y, Kawashima M, Furumichi M, Tanabe M (2016). KEGG as a reference resource for gene and protein annotation. Nucleic acids research.

[22] Yu G, Wang LG, Han Y, He QY (2012). clusterProfiler: an R package for comparing biological themes among gene clusters. Omics: a journal of integrative biology.

[23] (2015). Gene Ontology Consortium: going forward. Nucleic Acids Res.

[24] Almaani S, Meara A, Rovin BH (2017). Update on Lupus Nephritis. Clin J Am Soc Nephrol.

[25] Hakkim A, Fürnrohr BG, Amann K, Laube B, Abed UA, Brinkmann V, Herrmann M, Voll RE, Zychlinsky A (2010). Impairment of neutrophil extracellular trap degradation is associated with lupus nephritis. Proc Natl Acad Sci U S A.

[26] So BYF, Yap DYH, Chan TM (2021). MicroRNAs in lupus nephritis-role in disease pathogenesis and clinical applications. Int J Mol Sci.

